# Surgical pathway for HIV‑infected patients based on the ERAS strategy (Review)

**DOI:** 10.3892/mi.2024.174

**Published:** 2024-07-09

**Authors:** Wei Mao, Xinhua Jiang, Xilin Zeng, Deqiang Ye

**Affiliations:** 1 Department of General Surgery, Nanchang University Infectious Disease Hospital, Nanchang, Jiangxi 330002, P.R. China; 2 Department of Anesthesiology, Nanchang University Infectious Disease Hospital, Nanchang, Jiangxi 330002, P.R. China

**Keywords:** human immunodeficiency virus, enhanced recovery after surgery, acquired immunodeficiency syndrome, surgery, management

## Abstract

Enhanced recovery after surgery (ERAS), which is based on evidence-based medicine, focuses on patients and aims to reduce the psychological and physiological trauma stress reactions and complications of patients, thus shortening the duration of hospitalization, promoting rapid recovery and reducing medical expenses, readmission rate and mortality rates. Acquired immunodeficiency syndrome (AIDS) is caused by human immunodeficiency virus (HIV) infection. Patients with HIV/AIDS, as with other patient populations, can suffer from several surgical-related diseases. Therefore, the need for surgery in this group of patients exists and the surgical services required by patients with AIDS has gradually become an urgent matter of concern. According to relevant literature and the authors' clinical experience, the present review summarizes the current surgical approaches for patients infected with HIV based on ERAS. In the present review, the related issues observed at different stages of surgery, including pre-operative, intra-operative, post-operative and follow-up stages, are discussed.

## 1. Introduction

Enhanced recovery after surgery (ERAS) strategies aim to reduce the psychological and physiological trauma stress reactions of patients, based on current evidence-based medical findings. ERAS can optimize particular peri-operative treatment strategies via the close cooperation of surgery, anesthesia, nursing and nutrition, to prevent peri-operative stress reactions and complications, decrease the duration of hospitalization, promote rapid recovery and reduce medical expenses, as well as readmission and mortality rates. This novel strategy is commonly applied throughout the peri-operative period and is involved in highlighting the concept of patient-centered diagnosis and treatment (1).

In 1997, the Danish scholar, Kehlet, first proposed the concept of fast track surgery (FTS) and attracted worldwide attention (2). After 4 years, Europe established the ERAS study group and formally renamed FTS to ERAS (3). In 2010, the ERAS Association was established in Europe and several clinical guidelines were then issued (4,5). In 2013, the ERAS Society was also established in the USA (6). In 2007, a famous Chinese academician, Professor Jieshou Li, took the lead in introducing ERAS into the Chinese mainland and applying it to clinical practice (7). He also released an expert consensus on a variety of diseases between 2016-2019 (8,9). Following >20 years of clinical research and promotion, ERAS is currently considered a novel discipline focusing on peri-operative optimization (10).

Acquired immunodeficiency syndrome (AIDS) is caused by human immunodeficiency virus (HIV) infection. Since the first case of AIDS was reported in the 1980s, AIDS has been a major public health concern worldwide (11). Several studies at home and abroad have suggested that the number of reported cases of HIV infection or AIDS (HIV/AIDS) is increasing annually worldwide (12-14). With the continuous improvement of living standards and medical conditions, the average lifespan of the worldwide population is gradually increasing. Consistently, the AIDS population is also gradually exhibiting an aging trend (13,15). With the development and progress of anti-retroviral therapy, AIDS has gradually become a chronic disease that can be effectively controlled for a long period of time. Therefore, the survival time of the infected population has also been prolonged. As of 2018, there are an estimated ~1.25 million HIV/AIDS cases alive in China (14). Among these, the proportion of patients aged >50 years is increasing annually (16). AIDS research on the elderly has become a novel trend of research at home and abroad. HIV invasion into the human immune system gradually leads to impaired human cellular immune function, eventually rendering the human body susceptible to various opportunistic infections and tumors. In addition, patients with HIV/AIDS, as with other disease populations, can suffer from several other surgical-related diseases. Therefore, surgery in this group of patients is often necessary and therefore, the surgical services required by patients with AIDS has gradually become an urgent matter of concern (17,18).

ERAS not only focuses on the treatment of various surgical-related diseases, but also on ensuring the speed and quality of the post-operative rehabilitation of patients. The goal of ERAS is to minimize the pain and risk of patients (19,20). According to relevant literature and the authors' clinical experience, the present review article summarizes the current surgical approaches applied in patients infected with HIV based on the ERAS strategy.

## 2. Pre-operative stage

### Admission evaluation

The majority of patients can undergo surgery within 3 days following hospital admission. Therefore, the relevant auxiliary examinations should be completed as soon as possible following admission. Furthermore, the pre-operative evaluation should be also completed based on the results of the auxiliary examinations. As opposed to other patients, individuals infected with HIV need to be assessed for immune system function, psychological status and cognitive function (21).

*Collection of basic admission information.* The nationality, occupation, marital status, education level, working status, living status, health status, family composition and family economic status should be first recorded in detail. The clinicians should complete necessary auxiliary examinations, including blood, urine and stool routine tests, coagulation, liver, kidney, cardiopulmonary and thyroid function assessment, electrolyte levels, chest X-ray, electrocardiogram, ultrasonography and computed tomography (CT) and magnetic resonance imaging (MRI) scans of the sites of interest, as soon as possible. In addition, attention should be paid in the detection of particular infectious diseases, such as viral hepatitis, syphilis and tuberculosis (21).

*Medical history.* The clinicians should record any history of drug or food allergy, a history of surgery, alcohol addiction, drug abuse, complications, the administration of anticoagulants or other drugs, as well as the risk of the patient developing venous thrombosis, and cardiovascular and cerebrovascular diseases (21).

*Specific examinations.* Furthermore, several specific examinations should be carried out to verify whether the disease had been well-diagnosed and whether further examinations, including invasive examination and surgery, are required. When surgery is necessary, the duration of surgery and method, anesthesia, resection range and intra-operative conditions should be carefully estimated and prepared. The specific equipment required during the surgery should be prepared and a post-operative treatment plan should be prepared in advance (21).

*Respiratory system management.* Respiratory system management is crucial in the ERAS strategy. For example, smoking can reduce the ability of tissue oxygenation, thus leading to an increased risk of incision and pulmonary infections, as well as venous thrombosis following surgery. Therefore, smokers should stop smoking for >2 weeks prior to surgery. In addition, patients with respiratory dysfunction should take some functional exercise. It has been reported that several patients with HIV are prone to opportunistic infections, such as *Pneumocystis carinii-*mediated lung infection, tuberculosis and *Penicillium marneffei* infection. Therefore, clinicians should pay attention to identify and treat these infections prior to surgery (22,23).

*Psychological assessment.* In terms of psychological status, clinicians should evaluate the social interpersonal association, disease-related stress, emotional and mental state of patients, and whether personality traits are impulsive or despair-filled, as well as whether active antiviral therapy should be administered (24,25).

*Immune function evaluation.* CD4^+^ and CD8^+^ T-lymphocyte counts and plasma HIV load, should be measured prior to surgery. Therefore, effectively reducing the viral load and increasing the CD4^+^ T-lymphocyte count of HIV-infected patients during the peri-operative period is of utmost importance for reducing surgical complications, as well as the risk of surgery and occupational exposure of medical workers (26,27). When the number of CD4^+^ T-lymphocytes in patients is ≥350/µl, the peri-operative treatment should be the same as that applied in other patients. When a viral load of ≤200/µl and a CD4^+^ T-lymphocyte count of <350/µl are recorded, the surgery should focus on limiting surgical injury. Additionally, when a CD4^+^ T-lymphocyte count of <200/µl is detected, the surgery should be performed very cautiously. For HIV-infected patients undergoing elective surgery, it is recommended to increase the number of CD4^+^ T-lymphocytes prior to the surgery. However, for patients who do not need to undergo emergency surgery, their viral load prior surgery should be reduced to a targeted level, and they and their families should be fully informed of the risks involved and decide whether they will undergo the surgery or not (21,28).

*Assessment of cognitive function.* It has been reported that the incidence rate of HIV-associated neurocognitive disorders (HAND) is as high as ~50% (29). In China, the incidence rate of HAND among HIV-infected individuals is estimated to be ~40%. Therefore, HAND has become one of the main causes of mortality in HIV-infected subjects (30). The most commonly used tools for assessing cognitive function are the Montreal Cognitive Assessment (MoCA) scale and the International HIV Dementia Scale (IHDS). HAND can be divided into the following three levels: Asymptomatic neurocognitive impairment, mild cognitive disorder and HIV-associated dementia (31). Therefore, patients infected with HIV should be evaluated for HAND prior to surgery. If necessary, the appropriate drugs should be selected to improve the symptoms of patients with HAND (32).

*Surgery risk assessment*. In HIV-infected individuals, immune function can be further impaired and infection-related complications can increase following surgery. Liu *et al* (33) designed an operation risk scoring table for HIV-infected subjects to comprehensively evaluate the surgery risk. Therefore, according to the scores of the CD4^+^ T-lymphocyte count, incision classification, surgical grading, the presence of non-communicable infections and the presence or absence of organ dysfunction, the incidence of post-operative sepsis can be only 10%, when a score of ≤9 is obtained. However, when a score of ≥10 is recorded, the incidence of sepsis can reach 90% (33).

### Professional knowledge education and psychological counseling

The majority of patients with HIV lack professional knowledge of the disease and are prone to anxiety and depression. Therefore, professional knowledge education and psychological counseling are crucial, since with the trust of patients, anxiety and depression can be relieved, and can be better coordinated with treatment (34).

### Cooperate with anesthesiologists to complete pre-operative visits

Prior to surgery, anesthesiologists should record the height and weight of the patients, complete an assessment of cardiovascular, cerebrovascular, venous thrombosis and anesthesia risk, and select the appropriate anesthesia methods and anesthetic drugs. Additionally, the education of anesthesia professional knowledge and relevant psychological counseling should be provided. The following points should be considered during the pre-operative anesthesia visit of HIV-infected patients: i) Routine laboratory test results; ii) electrocardiogram and echocardiography examinations, when necessary; iii) pulmonary function test and arterial blood gas measurement; approximately two-thirds of patients with HIV suffer from respiratory system-related diseases during their illness; therefore, the screening of recessive lung diseases is of utmost importance; iv) X-ray and CT chest radiography, if necessary; v) when demyelinating lesions are suspected, spinal or brain MRI scans should be performed; opportunistic brain infections, such as microsporiasis and non-specific ones, can be detected on MRI and CT scans; vi) if HAD is reported, the consent of family members should be obtained prior to surgery; vii) CD4^+^ T-lymphocyte count; viii) patients with a history of drug abuse can experience difficulty in receiving intravenous injections; ix) the interaction between drug abuse and narcotics should be also considered; and x) the mental state of the patients (21).

### Pre-operative nutrition evaluation and supportive treatment

Prior to surgery, whether malnutrition is present and the cause of it should be also determined. Therefore, it is necessary to evaluate whether nutrition should be strengthened, as well as to select the appropriate supplementary nutrition approach. When patients undergo a weight loss of >10% of their body weight within a period of 6 months, and have a nutritional risk screening score of >5, a body mass index of <18.5 kg/m^2^ and serum albumin levels of <30 g/l, they should be considered to be at a severe nutritional risk. Therefore, these patients should receive the appropriate nutritional support, with enteral nutrition being the first choice (35).

### Preparation of patients the night prior to surgery and on the morning of the day of the surgery

According to the surgical plan, the clinicians should verify whether intestinal preparation, diet adjustment, preoperative fasting, skin preparation, gastric tube retention and urinary catheter are required. The quality of the patient's rest the night prior to the surgery should be guaranteed, while sedatives, such as benzodiazepines, can be used as appropriate (21).

## 3. Intra-operative strategy

### Anesthesia

Previous research has demonstrated that there is no difference between HIV-infected individuals and other patients in terms of their tolerance to general anesthesia when undergoing surgery during the asymptomatic period (36). The tolerance of HIV-infected subjects to general anesthesia is associated with the presence of other complications and the degree of AIDS development, but not with the HIV infection per se. Therefore, emerging evidence has suggested that the application of general anesthesia in HIV-infected individuals is safe and feasible (37,38). However, a previous study demonstrated that the cellular immune function, including the activity of natural killer cells, T-lymphocytes, monocytes and neutrophils, was further impaired following general anesthesia (39). General anesthetics-mediated immunosuppression can occur within 15 min following the induction of anesthesia and can last for 3-11 days. Although anesthesia drug-mediated immunosuppression can exhibit minimal clinical significance in healthy individuals, it can affect HIV-infected patients with immunodeficiency (40). According to a previous study, the majority of HIV-infected individuals were asymptomatic and/or treated with highly active antiretroviral therapy for >5 years. They also had a CD4^+^ T-lymphocyte count of >200/µl. In daily anesthesia practice, HIV-infected patients with AIDS can be at different stages of the disease, while treatment with ART can also affect their tolerability to anesthetic drugs (41). At the same time, the potential effect of HIV infection on each organ system should be also considered. Therefore, HIV-infected patients may be at a high risk of developing anesthesia-related complications and thus, more accurate peri-operative management and individualized anesthetic drugs are required.

### Anesthesia mode selection

In general, HIV-infected individuals do not require special anesthesia mode. Therefore, the type of anesthesia applied to these patients is commonly determined by the condition and type of surgery. Contraindications for intraspinal anesthesia were recorded in patients with HIV-related neuropathy, increased intracranial pressure and central nervous system infections (41). In addition, attention should be paid to the effects of HAND on anesthesia.

### Induction of anesthesia and endotracheal intubation management

Additionally, the airway condition should be also evaluated prior to the induction of anesthesia. Clinicians should also communicate well with patients to reduce their psychological tension. For particular patients, such as those with oropharyngeal and esophageal diseases, the difficulty and risk of tracheal intubation should be fully evaluated (41). It is recommended to use rapid induction of intravenous anesthesia to effectively inhibit the stress reactions in order to stabilize the condition of patients. Additionally, anesthesiologists should take effective protective measures, such as gloves and face masks, while the use of a visual laryngoscope and disposable intubation lens is highly recommended (41).

### Maintenance of anesthesia

For patients with AIDS, clinicians should carefully use narcotic drugs. It has been reported that antiretroviral therapy can affect cytochrome P450 levels (42). Therefore, there may be potential drug interactions. The metabolism of etomidate, atracurium, remifentanil and desflurane is independent of cytochrome P450 and these drugs are thus preferred. By contrast, the metabolism of midazolam and fentanyl can be affected by cytochrome P450 and should thus be avoided (43). Anesthetics and muscle relaxants should be used more reasonably in patients with AIDS-related anemia, fever, dehydration, hypoproteinemia, tachycardia and electrolyte imbalance (41).

### Monitoring of the depth of anesthesia

It is recommended to perform routine monitoring to control the particular depth of anesthesia, thus ensuring that the stress response during the surgery would be continuously and effectively suppressed. In this manner, the immunosuppression of HIV-infected patients could be alleviated and their post-operative recovery may be facilitated (41).

### Maintenance and management of the respiratory and circulatory system during surgery

Generally, there is no difference in terms of respiratory and circulatory system management during surgery between HIV-infected individuals and other patients, unless patients suffer from any other complications and thus require specific treatment. In the case that a patient suffers from a lung disease, increasing the oxygen concentration during the surgery may be necessary. Tachycardia is very common during anesthesia and can be improved gradually after surgery. However, tachycardia should be monitored and caution should be exercised in patients with myocardial ischemia, decreased cardiac function and hypoxia. Fever, anemia and tachycardia are more common among HIV-infected patients following surgery and attention should be paid to screen patients for secondary infections (40).

### Management if intraoperative temperature

The routine monitoring of body temperature, particularly in the cases of surgeries with a long duration, increased blood loss and large blood infusion volume, should be performed so as blood coagulation and the post-operative recovery of the patient is not affected (41).

### Anesthesia awakening and tracheal extubation

Prior the end of the surgery, the amount of drugs used for general anesthesia should be reduced, and patients should be treated with analgesic drugs. Additionally, to achieve smooth extubation, restlessness during awakening should be avoided. The anesthesia machine, monitor and other instruments should be strictly disinfected after surgery (41).

### Occupational exposure protection

The risk of cross infection in anesthesia practice should be reduced. Anesthesiologists can be infected by HIV via mucosal surface injury and body fluid splash, which could be caused by a sharp instrument. A previous study reported an average risk of needle stick injury and skin mucosal HIV transmission of 0.3 and 0.03%, respectively. Factors that increase the risk of infection after acupuncture injury include the amount of blood exposed, such as in the case of hollow needle injury, and the depth of acupuncture. The cumulative risk of HIV transmission during anesthesia can be as high as 4.5%. Therefore, clinicians should be vigilant when taking preventive measures. However, the infection control measures taken by anesthesiologists are still insufficient (44). It has been reported that wearing gloves can reduce the risk of needle stick injuries by 10-100 times (33). Following occupational exposure to HIV, it is recommended that the medical personnel should initiate post exposure prevention as soon as possible, preferably within 1-2 h following exposure. All hospitals should set up reasonable procedures to promote the implementation of the above post-event protective measures. Finally, the contamination of anesthesia equipment is a potential route of transmission between patients. Therefore, disposable equipment should be used wherever possible (33).

### Responsibility of clinicians

The responsibilities of clinicians include the following: i) Investigating surgery and determining the extend of resection; ii) identification and protection of key organs during the surgery; iii) the termination of bleeding accurately during the surgery and preventing post-operative bleeding; iv) fine operation and judicious use of new instruments to reduce injury can accelerate post-operative rehabilitation; v) the use of prophylactic antibiotics; patients with HIV infection have a poor immune function; thus, prophylactic antibiotics should be routinely used during the peri-operative period; for surgeries with greater scope of injury, the use of antibiotics should be extended appropriately and the dose should be also increased (45,46); patients who are at a high risk of developing sepsis should also receive antibiotic prophylaxis; vi) to be protected against occupational exposure to HIV, clinicians and the medical personnel should take special precautions, including isolation clothing, waterproof boots, double-layer waterproof surgical suits, double-layer gloves, goggles and face shield masks. In addition, adequate ambient lighting, orderly placement of objects and good management of sharp tools should be guaranteed (33).

## 4. Post-operative management

Post-operative bleeding at the area at which the surgery was performed should be carefully monitored. Additionally, post-operative throat pain, nausea, vomiting, dizziness, headache, incision pain and fever should be treated in time. Surgeons (or nurses) should explain the causes of post-operative complications to patients, alleviate their anxieties and timely provide them with the appropriate treatment when necessary. Following gastrointestinal function recovery, antiviral therapy should continue to be taken by patients in a standardized manner. Furthermore, early post-operative ambulation and functional exercise can promote the recovery of gastrointestinal and skeletal muscle functions, and reduce the risk of pulmonary infection, soft tissue pressure injury and venous thromboembolism, and should thus be considered. In addition, doctors and nurses or other professionals should guide patients to gradually resume their diet according to the particularity of their condition. Finally, post-operative immune function examinations, including the measurement of CD4^+^ and CD8^+^ T-lymphocyte count and plasma HIV load, should be routinely performed (33).

There are reports that due to impaired immune function, the incidence of complications, such as surgical site infections in HIV-positive patients is significantly higher than that in HIV-negative patients (47). Therefore, the prophylactic use of antibiotics is necessary during the peri-operative period. For patients who require a lengthy surgical procedure, or those with an advanced age and potential health risks, the use of antibiotics should be appropriately extended and higher-level antibiotics should be selected. In particular, in patients with high-risk factors for surgical site infections, the prophylactic use of antibiotics is necessary. When the immune function is poor, antifungal drugs should be properly used to prevent and treat fungal infectious diseases such as pneumocystis pneumonia (21).

## 5. Follow-up after discharge

Patients with a normal immune function can refer to the discharge conditions of ordinary patients. A limited number of patients can undergo daytime surgery if their condition allows it (48). The monitoring of immune function after discharge should be continued, including the routine measurement of the CD4^+^ and CD8^+^ T-lymphocyte count and plasma HIV load. Patients should be followed-up regularly by visiting the clinic, by phone or by other forms (41).

The implementation of ERAS for HIV-infected patients assumes that the medical personnel should follow rules and evidence for dealing with such patients, which is of utmost clinical significance. In 2014, a medical unit, particularly for patients with infectious diseases who need to undergo surgery, was established at the Nanchang University Infectious Disease Hospital (Nanchang, China). This unit carries out ~170 surgeries on patients with AIDS each year. Currently, under the guidance of ERAS, satisfactory results have been achieved. Patient satisfaction has gradually increased from 91.3% in 2014 to 99.4% in 2022, while none of the medical staff in this surgical team experienced occupational infections.

## 6. Conclusions and future perspectives

When HIV-infected patients are scheduled to undergo surgery, a thorough pre-operative evaluation and supportive treatment should be provided. During the surgery, anesthesia should be managed accordingly and reasonably, while the medical staff should be cautious to avoid HIV infection. Following surgery, patients should be intensively monitored and treated effectively as necessary.

## Data Availability

Not applicable.
